# Size estimation of injecting drug users (IDU) using multiplier method in five Districts of India

**DOI:** 10.1186/1747-597X-7-9

**Published:** 2012-02-21

**Authors:** Gajendra Kr Medhi, Jagadish Mahanta, Brogen S Akoijam, Rajatashuvra Adhikary

**Affiliations:** 1 Regional Medical Research Centre, NE Region, Indian Council of Medical Research, Dibrugarh 786001, Assam, India; 2 Community Medicine Department, Regional Institute of Medical Science, Lamphelpat, Imphal 795004, Manipur, India; 3 Program Sciences, FHI 360, 1825 Connecticut Avenue, Washington DC 20009, USA

## Abstract

The HIV epidemic in Manipur, the highest HIV prevalence state of India, is primarily driven by injecting drug use. Reliable estimate of population size of injecting drug users (IDU) is critical for aiding HIV prevention program in the state to combat drug driven HIV epidemic. The study described multiplier method, an indirect technique of estimation of IDU size in five districts of Manipur, India making use of existing records of rapid intervention and care (RIAC) programs. Number of IDUs who accessed RIAC services during the past 12 months was taken as the benchmark data for the size estimation. The benchmark data were then multiplied by the inverse of the proportion of the IDUs who reported having accessed RIAC services during the same period to derive the sizes of IDU population in each study districts. The estimated sizes of IDU population in five districts were: 7353 (95% CI: 6759-8123) in Imphal West, 5806 (95% CI: 5635-6054) in Imphal East, 3816 (95% CI: 3571-4139) in Thoubal, 2615 (95% CI: 2528-2731) in Churachandpur and 2137 (95% CI: 1979-2343) in Bishenpur district. Multiplier method seems to be a feasible indirect technique which can be applied to estimate of IDU population using existing data from intervention programs in settings like Manipur where reliable size estimation of IDU population is lacking.

## Findings

Injecting drug use is a global public health problem that contributes significantly to the transmission of bloodborne viral pathogens, including HIV and is responsible for an increasing proportion of new HIV infections [1-4]. Approximately 10% of the HIV/AIDS cases worldwide are attributed to injecting drug use (IDU) [4]. In India, injecting drug abuse is an important driving force of HIV epidemic in its northeastern states bordering Myanmar. Manipur is the most affected state by the injecting drug abuse-related HIV epidemic and has the highest HIV prevalence in India [5]. According to the latest HIV sentinel surveillance report, the prevalence of HIV among IDUs in the state is about 18% [6,7]. Reliable estimation of the size of IDU population is essential for policy advocacy, allocating resources for prevention interventions, treatment and care, estimations and projection of numbers of persons infected with HIV, and monitoring and evaluation of interventions [8].

However, despite its critical importance, reliable size estimates of drug injecting population have received little attention in this region. Societal stigma and hostility associated with illegal drug use in the region often force the IDUs to hide their identity as a drug user [7,9]. The hidden nature of IDUs makes it nearly impossible to estimate population size using direct counting methods such as census [8]. Therefore, it is necessary to resort to indirect techniques to arrive at estimates of size of such hidden populations at high risk for HIV. Various indirect methods for estimating population size of such hidden populations have been proposed [8,10-12]. Capture-recapture (CRC) methods are perhaps the most frequently used method of size estimation. But, CRC is harder to implement because of methodology involving matching of individuals. Further, the CRC methods also require various assumptions that limit its application in estimating population size. Multiplier is an alternative indirect approach that is mathematically simple, straight forward and relatively easy to implement with proper preparation [8,10,11]. Unlike CRC methods, it is not necessary to match individuals in the multiplier method. Of all the indirect methods of size estimations, multiplier approach is probably the easiest to implement [10]. However, very few examples of the use of multiplier methods in estimating size of populations at risk for HIV have been recorded in the scientific literature [8]. The key issue in using multiplier method is to find two independent data sources that overlap in known ways [8]. Multiplier approaches to estimate population size of female sex workers (FSW) or men having sex with men (MSM) have been made in other settings combining program/institutional data and behavioural survey data of target population [12,13]. Availability of IDU registries maintained by intervention programs in Manipur also provides an opportunity to use this multiplier method for estimating the size of IDU population in this state. We therefore carried out a study in Manipur to describe the use of multiplier method to estimate the size of IDU population from the available program data. We are describing our experiences, difficulties and challenges in estimating size of IDU population in five districts of Manipur employing this indirect technique.

Multiplier method estimates the population size using information from two independent data sources that overlap in a known way [8,11]. One data source is usually an institution or intervention program (known as benchmark population), which the target population is in contact with, and the other is the survey of target population itself. To use program/institutional and survey data together to estimate the size of a population, the members of the population all have to have a chance of being included in both the survey and in the institutional or program data [8,11]. Estimates can be derived by multiplying the numbers of persons who attended or accessed selected institutes or services over a specified time frame by the inverse of proportion of the population who say they attended or accessed the services over the same time period [8,14]. Based on the above principle, the population size of the IDUs can be estimated using the formula- (N = (1/P) × M, where, N is the size of drug injecting population being estimated, M is the benchmark data i.e. total numbers of IDUs who attended or received services in a given period from a specific institute/program, P is the proportion of IDUs in the study area who reported having attended or received the institute/services in the same period. In this study, institutional data/benchmark data (i.e. M) was obtained from the ongoing rapid intervention and care (RIAC) programs in each study districts. On the other hand, the proportion of IDUs accessing the services from the RIAC centres (i.e. P) were estimated from an independent survey of the IDU population in each study districts in order to calculate the multiplier (i.e. 1/P). There are two elements in the multiplier method. First one is the 'benchmark' (M) data which represents the known portion of IDUs who have accessed services of intervention programs. The second element required by the method is a multiplier that tells how many more injecting drug users in the specific districts have not accessed RIAC services in the last 12 months. That multiplier (1/P) can be worked out simply if the proportion of IDUs in the district accessing RIAC services during the same period can be calculated or determined as described above [10].

We carried out the study in five districts i.e. Imphal East, Imphal West, Thoubal, Bishnupur and Churachandpur. Apart from Churachandpur district that is hilly, other 4 districts are situated in plain terrain. Churachandpur is the largest and Imphal West is the most populous among the 5 districts selected for the study (Table 1) [15]. These 5 districts were selected because the RIAC programs maintained IDUs registries in these districts. Males aged 18 years or older belonging to a specific district, who injected drugs for non-medical purposes at least once in the past 6 months were defined as IDU in the study. Ethical approval for conducting the study was given by institutional ethical committee of Regional Medical Research Centre (RMRC) of Indian Council of Medical Research (ICMR). All the Interviews were conducted employing unlinked and anonymous procedure. No interviews were conducted without obtaining prior informed consent from the participants.

**Table 1:** Population and land area of the study districts

District	Total Populations	Land Area (km^2^)
Imphal East	394876	469.4
Imphal West	444382	519
Thoubal	364140	514
Bishnupur	208368	496
Churachandpur	227905	4570

Population size estimation was done separately for each study districts. The benchmark data (institutional or service provider data) used for each study districts were numbers of IDUs who received services from different RIAC centres within last 12 months and were obtained from RIAC centres through the Manipur AIDS Control Society (MACS). The benchmark figure was arrived at by combining the data of different RIAC centres. To avoid data duplication, those IDUs accessing services from outside the areas covered by a particular RIAC center were excluded from the benchmark figure. Further exclusion of IDUs receiving RIAC services from outside the study district (i.e. catchments area) were also made from the benchmark figure. Thus, a final combined revised list of those IDUs accessing services of RIAC centres in the last 12 months was prepared for each study districts in consultation with different RIAC centres.

We also conducted separate anonymous interviews of IDUs to obtain information about exposures to RIAC services in the last 12 months in the month of September 2004. For obtaining IDU samples, mapping of IDU locations (hot-spots) where IDUs congregated for injecting, buying and hanging out in all the districts were carried out. Details methodology of mapping of hot-spots has been described earlier [7]. In short, snowballing technique was used to identify the hot-spots. People acquainted with IDUs (e.g. outreach workers and other persons from NGOs associated with IDU intervention programs, current and ex-IDUs) acted as key informants (KI) to facilitate contact with IDUs. After mapping the hot-spots, approximately 50% of hot-spots in each district were chosen randomly for the selection of respondents for interviews. We included all the hot-spots in Thoubal district as numbers of hot-spots were few. Within the hot-spots, respondents were selected randomly from among the eligible IDUs available in the hot spots during the time of our field team visit. Approximately 50% of IDUs were selected from each selected hot-spots for interviews. A structured interview schedule containing 5 main questions was used for the interview. The question to determine exposure to intervention was-"*Have you accessed any services from any of the RIAC centres of the district (name of the district) at least once in the last 12 months*?". Exact binomial 95% confidence interval (CI) for proportions was calculated using Epi-info 6 software (Centers for Disease Control and Prevention, USA).

The findings of the study are presented in Table 2. The number of IDUs who received RIAC services in the last 12 months as per RIAC registry record varied from 1488 in Bishenpur to 5388 in Imphal East districts. During the survey, high proportion of IDUs (ranging from 65.9% in Bishenpur to 92.8% in Churachndpur) reported receiving RIAC services during last one year. The estimated population size was as follows: 7353 (95% CI: 6759-8123) in Imphal West, 5806 (95% CI: 5635-6054) in Imphal East, 3816 (95% CI: 3571-4139) in Thoubal, 2615 (95% CI: 2528-2731) in Churachandpur and 2137 (95% CI: 1979-2343) in Bishenpur district.

**Table 2:** Size estimation using multiplier method in 5 districts

District	IDUs attending RIAC centres within last 12 months (M)	Numbers of IDUs interviewed in the survey	Numbers of IDUs reported accessing RIAC service within last 12 months	Proportion of IDUs accessing RIAC services (95% CI)	Size of IDU population (95% CI)
Imphal East	5388	264	245	92.8 (89.0-95.6)	5806 (5635-6054)
Imphal West	4846	252	166	65.9 (59.66-71.7)	7353 (6759-8123)
Thoubal	2885	246	186	75.6 (69.7-80.8)	3816 (3571-4139)
Bishnupur	1488	250	174	69.6 (63.5-75.2)	2137 (1979-2343)
Churachandpur	2338	303	271	89.4 (85.6-92.5)	2615 (2528-2731)

M = Benchmark data (i.e. numbers of IDUs who received any services from RIACS in last one years as per registry record)

There are several important issues that should be kept in mind for estimating population size using multiplier methods. Multiplier is a mathematically simple and straightforward method, but it requires good quality institutional record keeping (i.e. benchmark data) for accurate estimation of population size [8,10,14]. In this study, numbers of IDUs accessing RIAC services in the last 12 months was taken as benchmark data. It was not possible from this study to determine the quality of records obtained from RIAC centres, but steps were taken to eliminate data duplication or to ensure that IDUs from outside the catchment areas were not included in the benchmark figure. The benchmark figure was arrived at by combining the records of multiple RIAC centres. Therefore, there was possibility of data duplication as IDUs under the coverage areas of one particular RIAC centre may access services from another RIAC centre. To eliminate such data duplication, IDUs accessing RIAC services from outside the area covered by a particular RIAC centre were subtracted from the benchmark figure. We believe that the IDUs were unlikely to provide false information about their names and addresses to peer/program workers of RIAC centres because of peer/program workers' closeness and familiarity with them. In multiplier method, the catchment area for the services or institutions should also be the same as that covered in the sub-population survey from which multipliers are derived [8,13]. In this study, those IDUs receiving RIAC services from out side the catchment area (i.e. from outside the study district) was also excluded from the benchmark as this information was also available from the RIAC centres.

Another important issue in multiplier method is the time reference period which must be clear and same in both data sources. In our study, the time reference period taken for the benchmark data was last 12 months. But, this time reference period used in benchmark was not consistent with the definition we used to define an IDU in the survey, where only those IDUs who had injected in the last six months were interviewed. Such issues of inconsistencies in the time reference period have also been reported in other studies [13]. But, this issue might have little effect on the size estimation if the proportion of IDUs who had quit their drug injecting career before six months period were low [13]. However, proper attention must be paid to the time reference issues in order to improve the accuracy of estimates in the future estimation.

For deriving an accurate multiplier, it is also essential to obtain reliable information about exposure to intervention from the respondents (IDUs) in the survey. In this study, we asked the respondents to report if they had accessed any services from RIAC centres in the last 12 months. Such longer time reference period (12 months) may however lead to recall errors of the events. Recall errors associated with longer time reference period can be minimized by shortening the time reference period. Apart from recall bias, self-reported information may also be subject to interviewer bias or socially desirable respond. To control such biases, interviewers were carefully selected and trained adequately by the investigators to build adequate skills among them to collect objective data from the respondents. In this study, 66% to 93% of IDUs reported receiving services from intervention program. These findings of this study were comparable to those found in another behavioural survey conducted among IDUs in Churachandpur and Bishenpur districts of Manipur in 2006 supporting the veracity of our results [16]. Report of National AIDS Control Society (NACO) in 2006 also indicates that high proportion (76%) of IDUs in Manipur received interpersonal communication on HIV/STI from interventional programs in the last year [17]. An IDU size estimation study carried in the Churachandpur district of Manipur in 2006 using CRC method found an estimate of IDUs which was comparable to the estimates of this study. That was the only IDU size estimation study from Manipur that may be helpful in validating the estimate from this study [14]. We found that self-reported proportion IDUs accessing RIAC services in Imphal East (92.8%) was much higher compared to adjoining Imphal West district (65.9%). Such variations in the service access from RIAC centres could be attributed to availability of relatively more service providers in the East district compared to the West.

Drawing a representative and random sample of IDU population is also a big challenge because of hidden nature of IDU population. However, to get representative samples of the study population and also to achieve better coverage of catchment area, we carried out an exhaustive mapping of IDU hot-spots and then respondents were selected from those hot-spots through a random selection process. Random selection of IDUs within the mapped hot-spots also helped in preventing self-selection biases or volunteerism. However, there may still be some biases in the approach adopted in this study [7]. Therefore, the study participants may not be representative of the entire study target populations because a bulk of IDUs may not often congregate at identifiable locations [7]. Some recent studies have therefore preferred respondent driven sampling (RDS) over other sampling methods to overcome such limitations for sampling such hidden population in this region [9].

To the best of our knowledge, this was the first study to estimate the size of IDU population using multiplier method in Manipur. This study revealed that estimation of population size of IDUs based purely on records of intervention programs does not represent the true size of the population as our study indicates that a sizeable proportion of IDU population remain unexposed to the intervention programs. Therefore, despite limitations, multiplier method may be used as a simple indirect tool to estimate size of IDU population utilizing the existing program data. Because of availability of IDU registries maintained by the intervention programs, the method seems to be sustainable in settings like Manipur. However, there are several key caveats which should be taken into account while using multiplier method for estimating the size of such hidden population. Understanding of some these caveats may help in the further application of this method using more recent data.

## Competing interests

The authors declare that they have no competing interests.

## Authors' contributions

GKM, JM and RA contributed in the conception of study design, oversaw the data acquisition, led data analysis, and drafted the manuscript. BSA contributed in data acquisition, study design, interpretation of the data and reviewing the manuscript. All the authors read the manuscript and approved the final version of the manuscript.
